# Oral health behaviour, attitude towards, and knowledge of dental caries among mothers of 0‐ to 3‐year‐old children living in Kaunas, Lithuania

**DOI:** 10.1002/cre2.272

**Published:** 2019-12-13

**Authors:** Sandra Petrauskienė, Julija Narbutaitė, Aušra Petrauskienė, Jorma I. Virtanen

**Affiliations:** ^1^ Clinic for Preventive and Paediatric Dentistry Lithuanian University of Health Sciences Kaunas Lithuania; ^2^ Department of Preventive Medicine Lithuanian University of Health Sciences Kaunas Lithuania; ^3^ Department of Clinical Dentistry University of Bergen Bergen Norway; ^4^ Medical Research Center Oulu University Hospital Oulu Finland

**Keywords:** child, ECC, mother, oral health attitude, oral health behaviour

## Abstract

**Objectives:**

This study aimed to investigate the oral health behaviours of mothers with young children and their attitudes towards dental caries.

**Methods:**

The survey targeted all mothers with children under 3 years attending a primary healthcare centre (Department of Family Medicine at the Lithuanian University of Health Sciences [LSMU] Hospital) in Kaunas, Lithuania. The Bioethics Centre of the LSMU approved the study (No. BEC‐OF‐14). Of 176 mothers, 123 (69.9%) took part in the 2016–2017 study. The self‐administered questionnaire enquired about mothers' attitudes towards oral health and behaviours related to the potential transmission of oral bacteria to their children, dietary habits, tooth brushing, smoking, and background factors. The chi‐squared test and univariate/multivariate logistic regression analyses served for the statistical analysis. (*p* values ≤ .05 indicated statistically significant differences).

**Results:**

Most (76; 68.5%) of the mothers brushed their teeth twice daily, and 97 (87.4%) reported themselves as nonsmokers. We found a statistically significant association between mothers who brushed their own teeth twice daily and those who cleaned their children's teeth likewise (OR = 5.42, 95% CI [1.28–6.63]; *p* = .005). We observed significant associations among mothers who gave their children sugar‐sweetened beverages (SSBs) daily and the mothers' college or lower education (OR = 6.51, 95% CI [1.59–27.19]; *p* = .01) and maternal tooth brushing less than twice daily (OR = 3.88, 95% CI [0.99–15.18]; *p* = .05).

**Conclusions:**

A majority of mothers who took part in this survey did not brush their children's teeth as recommended. Mothers with a lower education and who brushed their teeth less than twice daily offered their children SSBs more frequently.

**Abbreviations**
CI: confidence interval
ECC: early childhood caries
LSMU: Lithuanian University of Health Sciences
OR: odd ratio
SD: standard deviation
SSB: sugar‐sweetened beverages
SSP: sugar‐sweetened products

## 1 INTRODUCTION

Parents, and especially mothers, greatly influence their children's oral health as messengers of good health behaviour. Their greater understanding of oral hygiene and dietary habits also contributes to their children's oral health (Saied‐Moallemi, Virtanen, Ghofranipour, & Murtomaa, 2008). The children of parents with poor oral health are more likely to show poor oral health in adulthood than the children of parents with good oral health (Shearer et al., 2012).

Health behaviour comprises a complex variety of knowledge, attitudes, and behaviour, which all impact oral health (Virtanen, Vehkalahti, & Vehkalahti, 2015). Poor maternal oral health can significantly impact the general health of both the mother and her child and can, for example, increase the risk of developing caries in early childhood (Shearer et al., 2012). Mothers' health behaviours are associated with their practices towards their children's consumption of various sugar‐sweetened products (Laitala, Vehkalahti, & Virtanen, 2018). Mothers may also be sources of bacterial transmission to their children (Batra, Shah, & Virtanen, 2018; Virtanen et al., 2015). Some studies have reported a strong association between mothers' perceived oral health status and their children's oral health condition (Cademartori, Custodio, Harter, & Goettems, 2019).

Mothers' attitudes towards and knowledge of oral health directly affect their children's dental health outcomes; in contrast, insufficient knowledge of oral health, the main risk factors, and caries prevention can lead to inadequate counselling for children later (Batra et al., 2018; Tagliaferro, Ambrosano, Meneghim, & Pereira, 2008). Knowledge is one of the main risk indicators, and predictors of the disease make it possible to identify the individuals who would benefit most from early preventive measures (Tagliaferro et al., 2008). Oral health education targeting mothers may positively impact oral health status in children (Batra et al., 2018).

Oral hygiene plays an important role in the prevention of caries and periodontal diseases, and proper oral hygiene ought to be emphasised already from early childhood (Harris, Nicolli, Adait, & Pine, 2004). Oral hygiene habits are established in stages of development and are influenced by parental behaviours, predominantly of mothers, as they are often the primary caregivers of their children (Mohebbi, Virtanen, Murtomaa, Vahid‐Golpayegani, & Vehkalahti, 2008). Furthermore, when children are introduced to good health habits in early childhood, the good behavioural approach tends to continue later into adulthood (Batra et al., 2018).

Parental oral health‐related attitudes are also associated with children's oral health in both early childhood and later in life (Saied‐Moallemi et al., 2008). Parental awareness of their children's oral hygiene and their primary teeth status may play an important role in preventing future oral health problems (Batra et al., 2018). Oral health in preschool children is largely determined by behavioural factors, namely, inadequate oral hygiene habits, frequent consumption of sugar‐containing snacks and drinks, and lack of preventive visits to the dentist (Harris et al., 2004).

Studies have shown tooth brushing to be a good indicator of other habits affecting oral health. Both poor oral hygiene and high sugar consumption often occur in the same individuals (Tinanoff, 2017). Some parents believe that tooth brushing is the main caries‐preventive measure, whereas control of cariogenic eating habits has received less attention (Lenčová & Dušková, 2013). Researchers have found that behavioural dietary factors such as the extent, frequency, and time of sugar consumption relate more significantly to caries development than diet itself (Harris et al., 2004). Intake of sugar‐sweetened products begins in early infancy, and consumption frequency tends to increase among older children (Laitala et al., 2018). Patterns of sugar consumption change along the life course. The sense of taste changes, and independence in selecting food and beverages is higher among adults than among children (Sheehy et al., 2008).

Dental caries among children are common in Lithuania. Reports indicate that about half of 3‐year‐old children have early childhood caries (ECC) (Slabsinskiene et al., 2010). Bottle feeding, dietary habits, and lack of oral hygiene were the main determinants in ECC development. Up to 90% of 4‐ to 6‐year‐old children suffer from caries (Razmienė, 2013), leading to poor quality of life (Jankauskiene et al., 2014). Yet, research on maternal attitudes and behaviour towards oral health is scarce in Lithuania. We therefore aimed to investigate the oral health behaviours of mothers with young children and their attitudes towards ECC risk indicators.

## 2 STUDY POPULATION AND METHODOLOGY

The study targeted mothers with children under 3 years attending a primary healthcare centre in Kaunas, Lithuania. A self‐administered anonymous questionnaire (Virtanen et al., 2015) assessed the mothers' health behaviour and background information (Appendix S1). The Bioethics Centre of the Lithuanian University of Health Sciences approved the study (No. BEC‐OF‐14).

The study population comprised mothers with children under 3 years attending the Department of Family Medicine at the Lithuanian University of Health Sciences (LSMU) Hospital, the mid‐sized primary healthcare centre in Kaunas City providing free‐of‐charge services. With 300,000 inhabitants, Kaunas is the second largest town in central Lithuania. The most developed industries in Kaunas are the food and beverage, textile, and light industries. The majority (93.6%) of citizens are Lithuanians.

The Department of Family Medicine at the LSMU Hospital invites all mothers with children between 1 and 36 months for health check‐ups and vaccinations. About 200 mothers visit the centre annually. During routine visits for regular check‐ups and vaccinations for the children, the mothers were invited to voluntarily complete a self‐administered anonymous questionnaire distributed by the health nurses. To obtain a representative sample of mothers (approximately 200) from the health clinic, the estimated time needed to conduct the survey was 1 year. The survey was carried out from August 2016 to August 2017. During the mothers' routine visits to the clinic, two health nurses distributed the questionnaires to all and collected them immediately after the mothers completed them. All participants provided their written consent. Of 176 mothers visiting the centre, 123 completed the questionnaires (response rate: 69.9%). About 15% of registered children live abroad and do not pay routine compulsory visits. Fathers and grandparents with children paid regular visits to the public health clinic; mothers who refused to complete the questionnaire were excluded.

### 2.1 The questionnaire

The self‐administered questionnaire enquired about the mothers' background characteristics, perceived oral health status, smoking, attitudes towards oral health and behaviours regarding the potential transmission of oral bacteria to their children, the dietary habits of both the mothers and their children, such as the consumption of sugar‐sweetened beverages (SSBs) and sweets, and their own and their children's tooth brushing (Virtanen et al., 2015).

The first part of the questionnaire enquired questions about the mothers' oral health, tooth brushing habits, dental visits, smoking, and sweets consumption. The mothers' behaviour and understanding of their own oral health may impact their children's oral health behaviour (Virtanen et al., 2015).

The second part of the questionnaire enquired questions about the mothers' knowledge of and attitude towards indicators of dental caries risk (Virtanen et al., 2015). The questions were as follows: Gingivitis is caused by bacteria in the mouth; A sugary diet causes gingivitis; Frequent use of sugar increases dental decay; The use of fluoride prevents dental decay; Good oral hygiene inhibits tooth decay; Tooth decay is caused by too little use of fluoride toothpaste, frequent consumption of sugar, bacterial activity, characteristics of one's teeth, and how often one brushes one's teeth; Bacterial transfer from the mother's mouth to the child's mouth. Answer options used a Likert scale (*totally agree*, *partially agree*, *do not know*, *partially disagree*, and *totally disagree*). The question “Bacterial transfer from the mother's mouth to the child's mouth” had the following answer options: *never*, *seldom*, *quite often*, *often*, and *always*. The question “When should one start to brush one's child's teeth with fluoride toothpaste?” had several answer options: at eruption of the first tooth, when all primary teeth erupt, when permanent teeth start to erupt, and when child learns how to brush its teeth. The last question “At what age would it be good/beneficial to stop using a pacifier?” had the following answer options: until 1 year old, until 2 years old, until 3 years old, and as long as the child wants.

The third part of the questionnaire included questions about the mothers' behaviour towards their children's oral hygiene (one question), sweets consumption (two questions), and bacterial transmission from mother to child (four questions) (Virtanen et al., 2015). Inadequate oral hygiene, frequent sweets and SSB consumption, and possible transmission of cariogenic bacteria from mother to child are risk indicators of early childhood caries.

The mothers' background information included age in years (<25, 25–29, 30–34, 35–39, and 40+), later categorised into four by combining the two oldest age groups into one (35+). The mothers' level of education was categorised as basic, secondary, and college and university education, which was later combined into two groups: ≤college and university. Each child was classified into one of three age groups: 1–11 months, 12–23 months, and 24–36 months.

Questions about the mothers' tooth brushing had four options: never, almost every day, once a day, and more than once a day. These options were later dichotomised into two groups: less than twice daily and twice daily. Questions about tooth brushing of the children's teeth had the following options: not at all, seldom, once a week, every other day, once a day, and more than once a day. These options were later combined into four groups: never, less than daily (seldom, once a week, and every other day), once a day, and more than once a day (Laitala et al., 2018).

Questions about smoking had four options: smoke daily, smoke occasionally, do not smoke, and have quitted. All options were later dichotomised into the following groups: smoke daily/occasionally (smoker) and do not smoke/have quitted (nonsmoker).

Questions about the mothers' frequency of sugar‐sweetened products (SSPs) consumption (coffee or tea with sugar; other sweet drinks; biscuit, raisins or chips; candies) had five options (corresponding scores appear in brackets): more than three times per day (2), one to two times per day (2), two to five times a week (1), less frequently (0), and never (0). These scores were summed to describe the intensity of the mothers' SSP consumption and ranged from 0 to 8. The higher the score, the more frequent their reported SSP consumption. The scores for the intensity of the mothers' SSP consumption were categorised as low (scores 0–1), moderate (scores 2–6), and high (scores >6).

Questions about the children's consumption of SSBs and sweets/candies had six answer options (the corresponding scores appear in brackets): not at all (0), seldom (1), once a week (2), every other day (3), once a day (4), and more than once a day (5). The frequency of the children's SSB or sweets consumption was categorised as never, less than daily (seldom, once a week, or every other day) and daily (once a day or more than once a day). For further analysis, the original scores for SSB and sweets consumption were combined to assess the intensity of the children's SSP consumption. The scores for the intensity of the children's SSP consumption ranged from 0 to 10. The higher the score, the more frequent their total reported SSP consumption (Laitala et al., 2018). The scores for the intensity of the children's SSP consumption were categorised as low (scores 0–1), moderate (scores 2–6), and high (scores >6).

### 2.2 Statistical analysis

The Statistical Package for Social Sciences (SPSS version 22) served for the analysis. Chi‐squared tests served to measure differences between the mothers' background characteristics: smoking habits, their own health behaviour, and their oral health behaviour towards their children. We then calculated the means and standard deviations (SD); a *p* value ≤.05 indicated statistically significant differences.

Univariate logistic regression analysis, including the odds ratio (OR) and its confidence interval (95% CI), served to calculate the probability of an event (mothers giving SSBs or sweets/candies to their children, the frequency of their children's tooth brushing, sharing a spoon or mug/plate with their children, and cleaning their children's pacifier in their own mouth) corresponding to a certain risk indicator (the mother's education level, the frequency of the mother's tooth brushing, and kissing the child on the lips).

Multivariate logistic regression models, including the OR and its CI (95% CI), served in the complex evaluation of the probability of an event (giving SSB to a child daily), in light of certain indicators (mother's education level ≤college and the mother's tooth brushing less than twice daily).

## 3 RESULTS

The most prevalent age group of the mothers was 30–34 years 50 (40.7%), whereas mothers under 25 years comprised only 12 (9.8%) of the participants. The results revealed that a majority 77 (69.4%) of the mothers had a university education (Table 1).

**Table 1:** Characteristics and behaviours of the mothers (*N* = 123) of toddlers visiting the Family Medicine health clinic in Kaunas

Variables	%	*N*
Age of mother (year; missing *N* = 0)		
<25	9.8	12
25–29	22.8	28
30–34	40.7	50
35+	26.8	33
Mother's education (missing *N* = 0)		
≤College	30.6	34
University	69.4	77
Age of child (months; missing *N* = 0)		
1–11	26	32
12–23	32.5	40
24–36	41.5	51
Number of children in the family (missing *N* = 0)		
1	43.9	54
2	46.3	57
3+	9.8	12
Child care (missing *N* = 2)		
At home	73.6	89
Day care centre	26.4	32

The largest group of children was 24–36 months old 51 (41.5%), and 32 (26%) were 1–11 months old. The mean age of the children was 18.56 (10.0) months. Families with one or two children were the most common: 54 (43.9%) versus 57 (46.3%) in this survey (Table 1). Regarding child care, home care was significantly more prevalent among children under 36 months than care in day care centres (89 (73.6%) versus 32 (26.4%); *p* < .001).

Overall, a majority (97; 87.4%) of the mothers reported themselves as nonsmokers. In addition, significantly more mothers with a university education reported themselves as nonsmokers 72 (93.5%) than did mothers with a college or lower education 25 (73.5%), respectively (*p* = .003; Table 2). Considering the frequency of the mothers' tooth brushing, the results revealed that 76 (68.5%) of the mothers brushed their teeth twice daily. The mothers' education background showed no association with their tooth brushing behaviour. Although fewer mothers with a college or lower education 20 (58.8%) brushed their own teeth twice daily than mothers with a university education 56 (72.7%), the difference did not reach statistical significance (*p* = .146; Table 2).

**Table 2:** Mothers' (*N* = 123) own health behaviour and behaviour towards their child by education

Variables	Maternal education *N* (%)		Total *N* (%)	*p* value
	≤College	University		
Do you smoke? (missing *N* = 12)				
Daily/occasionally	9 (26.5)	5 (6.5)	14 (12.6)	.003
I do not smoke/have quitted	25 (73.5)	72 (93.5)	97 (87.4)	
Total *N* (%)	34 (100)	77 (100)	111 (100)	
How often do you brush your teeth? (missing *N* = 12)				
Less than twice a day	14 (41.2)	21 (27.3)	35 (31.5)	.146
Twice a day	20 (58.8)	56 (72.7)	76 (68.5)	
Total *N* (%)	34 (100)	77 (100)	111 (100)	
How often do you brush your child's teeth? (missing *N* = 15)				
Never	3 (9.1)	12 (16.0)	15 (13.8)	.048
Less than daily	9 (27.3)	15 (20.0)	24 (22.0)	
Once a day	8 (24.2)	34 (45.3)	42 (38.5)	
>Once a day	13 (39.4)	14 (18.7)	27 (24.8)	
Total *N* (%)	33 (100)	75 (100)	108 (100)	
How often does your child get sugar‐sweetened beverages? (missing *N* = 14)				
Never	7 (20.6)	27 (36.6)	34 (31.2)	.005
Less than daily	19 (55.9)	45 (60.0)	64 (58.7)	
Daily	8 (23.5)	3 (4.0)	11 (10.1)	
Total *N* (%)	34 (100)	75 (100)	109 (100)	
How often does your child get sweets/candies? (missing *N* = 13)				
Never	6 (17.6)	31 (40.8)	37 (33.7)	.05
Less than daily	26 (76.5)	40 (52.6)	66 (60.0)	
Daily	2 (5.9)	5 (6.6)	7 (6.4)	
Total *N* (%)	34 (100)	76 (100)	110 (100)	

*Note*: Chi‐square test, comparing results by maternal education (≤college and university).

In this survey, about one third of the mothers (42; 38.5%) brushed their children's teeth once daily, and one quarter (27; 24.8%) brushed their children's teeth twice daily. Among mothers with a young child (1–11 months old), about half (45.2%) reported not brushing their children's teeth, whereas 54% of those with a 24‐ to 36‐month‐old brushed their teeth twice daily (*p* < .001). More mothers with a university education (34; 45.3%) than mothers with a college or lower education (8; 24.2%) brushed their children's teeth once daily, whereas more mothers with a college or lower education (13; 39.4%) than mothers with a university education (14; 18.7%) brushed their children's teeth twice daily (*p* = .048); Table 2). Mothers brushing their own teeth twice daily showed a statistically significant association with those who cleaned their children's teeth likewise (OR = 5.42, 95% CI [1.28–6.63]; *p* = .005; Table 3).

**Table 3:** Mothers' behaviour towards their child in univariate logistic regression model

Characteristics	OR	95% CI	*p* value
Mother with college or lower education			
Giving sugar‐sweetened beverages for child			
Daily	7.385	1.82–29.97	.002
Less than daily	1		
Mother with university education			
Giving sweets/candies for child			
Yes	1		
No	3.215	1.191–8.681	.021
Mother's tooth brushing twice a day			
Frequency of child's tooth brushing			
Twice a day	5.42	1.53–19.24	.005
≤Once a day	1		
Sharing the spoon with the child			
No	2.91	1.28–6.63	.010
Yes	1		
Sharing the plate/mug with the child			
No	2.18	0.99–4.77	.049
Yes	1		
Pacifier cleaned in the mothers' mouth			
No	5.30	1.96–14.36	.001
Yes	1		
Mothers' tooth brushing less than twice a day			
Giving sugar‐sweetened beverages for child			
Daily	4.67	1.27–17.10	.013
Less than daily	1		
Kissing the child on the lips			
Giving sugar‐sweetened beverages for child			
Daily	3.07	1.36–6.92	.006
Less than daily	1		
Giving sweets/candies for child			
Yes	2.75	1.23–6.15	.012
No	1		

Abbreviations: CI, confidence interval; OR, odds ratio.

Considering the frequency of the children's SSB consumption, a majority (64; 58.7%) of mothers reported that their children consumed SSBs less than daily. A majority (71.9%) of mothers with a 1‐ to 11‐month‐old gave their children no SSPs (*p* < .001).

Significantly more mothers with a university education than mothers with a college or lower education (31 [40.8%] vs. 6 [17.6%], respectively) reported not giving sweets and candies to their children (*p* = .05; Table 2). In addition, significantly fewer mothers brushing their teeth twice daily than mothers brushing their teeth less than twice daily (4 [4.8%] vs. 7 [18.4%], respectively) reported giving SSB to their children daily (*p* = .039; Table 4).

**Table 4:** Mothers' (*N* = 123) behaviour towards their child by their own tooth brushing behaviour

Variables	Mothers' tooth brushing *N* (%)		Total *N* (%)	*p* value
	Less than twice a day	Twice a day		
How often do you brush your child's teeth? (missing *N* = 3)				
Never	8 (21.1)	10 (12.2)	18 (15.0)	.023
Less than daily	12 (31.6)	15 (18.3)	27 (22.5)	
Once a day	15 (39.4)	31 (37.8)	46 (38.3)	
>Once a day	3 (7.9)	26 (31.7)	29 (24.2)	
Total *N* (%)	38 (100)	82 (100)	120 (100)	
Do you feed your child with spoon, which you have used yourself for tasting? (missing *N* = 2)				
Yes	17 (45.9)	19 (22.6)	36 (29.8)	.010
No	20 (54.1)	65 (77.4)	85 (70.2)	
Total *N* (%)	37 (100)	84 (100)	121 (100)	
Do you feed your child from plate/mug, which you have eaten/drunken from? (missing *N* = 2)				
Yes	20 (52.6)	28 (33.7)	48 (39.7)	.049
No	18 (47.4)	55 (66.3)	73 (60.3)	
Total *N* (%)	38 (100)	83 (100)	121 (100)	
Do you have a habit to clean pacifier in your own mouth before giving it to the child? (missing *N* = 4)				
Yes	13 (36.1)	8 (9.6)	21 (17.6)	.001
No	23 (63.9)	75 (90.4)	98 (82.4)	
Total *N* (%)	36 (100)	83 (100)	119 (100)	
How often does your child get sugar‐sweetened beverages? (missing *N* = 2)				
Never	10 (26.3)	31 (37.4)	41 (33.9)	.039
Less than daily	21 (55.3)	48 (57.8)	69 (57.0)	
Daily	7 (18.4)	4 (4.8)	11 (9.1)	
Total *N* (%)	38 (100)	83 (100)	121 (100)	

*Note*: Chi‐square test, comparing results by mother's tooth brushing (less than twice a day and twice a day).

Mothers giving their children SSBs daily showed significant associations with mothers having a college or lower education (OR = 6.51, 95% CI [1.59–27.19]; *p* = .01) and maternal tooth brushing less than twice daily (OR = 3.88, 95% CI [0.99–15.18]; *p* = .05; Table 5). Mothers kissing their children on the lips associated with giving their children SSBs daily (OR = 3.07, 95% CI [1.36–6.92]; *p* = .013) as well as with giving their children sweets/candies (OR = 2.75, 95% CI [1.23–6.15]; *p* = .012; Table 3).

**Table 5:** Mothers' habit of giving SSB to their child daily in multivariate logistic regression model

Characteristics	OR	95% CI	*p* value
Educational level			
≤College	6.51	1.59–27.19	.010
University	1		
Twice‐a‐day tooth brushing			
Yes	1		
No	3.88	0.99–15.18	.05

Abbreviations: CI, confidence interval; OR, odds ratio; SSB, sugar‐sweetened beverage.

Overall, a majority (85; 70.2%) of mothers had no habit of sharing a spoon with their children (Table 4). Mothers' tooth brushing twice daily associated significantly with not sharing a spoon with their children (OR = 2.91, 95% CI [1.28–6.63]; *p* = .01; Table 3). Consequently, mothers brushing their teeth twice daily associated significantly with not sharing a plate or mug with their child (OR = 2.18, 95% CI [0.99–4.77]; *p* = .049) and not cleaning their child's pacifier in their own mouth before giving it to their child (OR = 5.30, 95% CI [1.96–14.36]; *p* = .001; Table 3).

**Figure 1:**
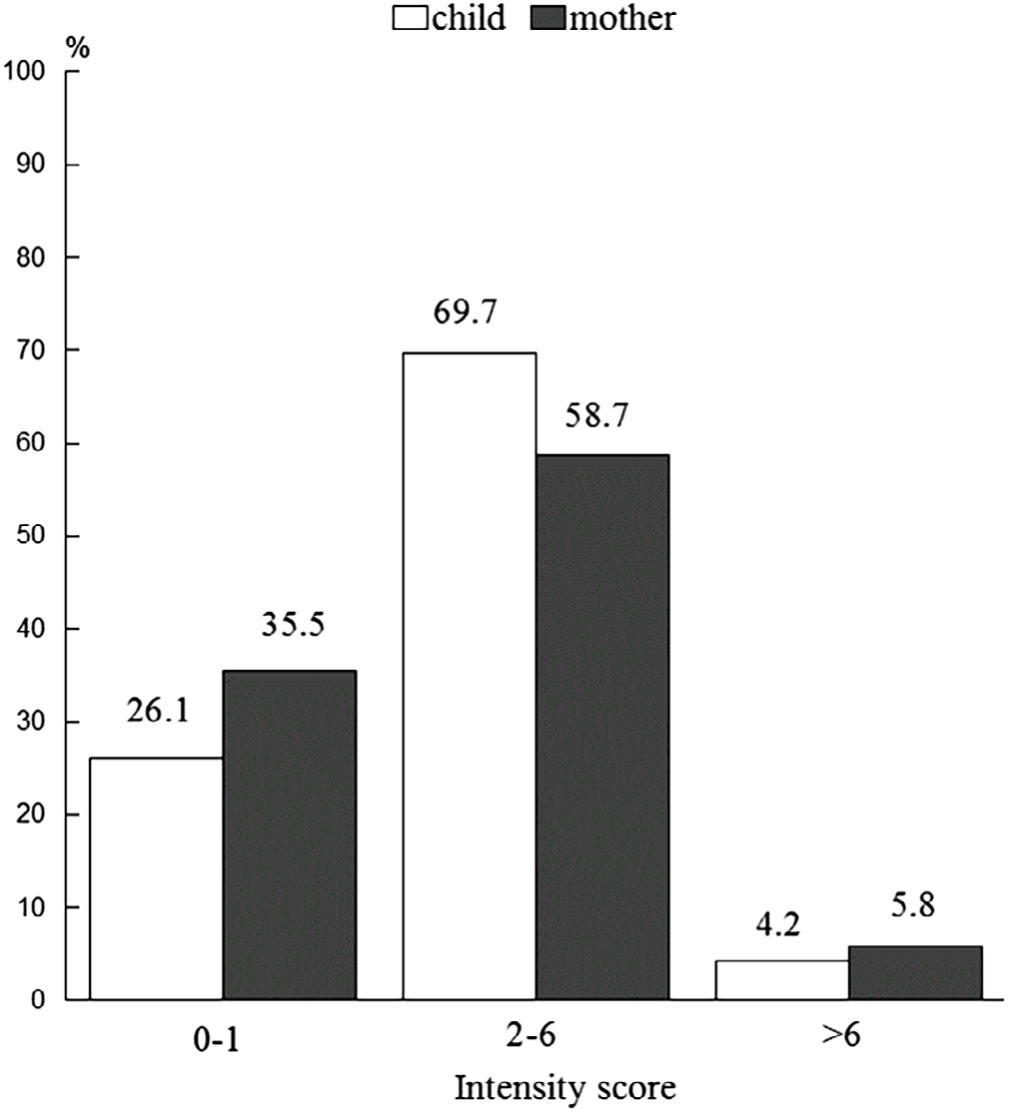
Distribution intensity scores of sugar‐sweetened product consumption by both mother and child

The scores for the intensity of the children's SSP consumption ranged from 0 to 10 (mean score: 2.64 [2.31]). Overall, 69.7% of the children consumed up to two different SSPs daily or less than daily. Subsequently, the scores for the intensity of the mothers' SSPs consumption ranged from 0 to 8 (mean score: 2.97 [2.09]). In sum, 58.8% of the mothers consumed up to three different SSPs daily (Figure 1).

## 4 DISCUSSION

Our study found that a considerable number, about one third, of Lithuanian mothers did not follow the universal recommendations for tooth brushing, and a clear majority of them did not brush their children's teeth as recommended. Mothers with a lower education and who brushed their teeth less than twice daily offered their children SSB frequently.

### 4.1 International comparisons

A number of studies have focused on parental oral health attitudes and behaviour towards children's oral health in order to prevent caries development in early childhood (Batra et al., 2018; Laitala et al., 2018; Lenčová et al., 2013; Virtanen et al., 2015; Watanabe et al., 2014). Parents feel responsible for their role in their child's tooth brushing and caries prevention but pay inadequate attention to their dietary habits (Lenčová 2013). Although tooth brushing twice daily is widely recommended (American Academy of Pediatric Dentistry, 2016), our study showed that, as in many other countries, a majority of the mothers cleaned their young children's teeth once a day or less (Batra et al., 2018; Laitala et al., 2018). More efforts ought to focus on improving tooth brushing habits in Lithuania, as in Finland, for instance, where a majority of mothers reported optimal behaviour, such as tooth brushing twice daily and not smoking (Virtanen et al., 2015).

In line with a recent Finnish study showing a weak correlation between mothers' smoking and their practice towards their children (Laitala et al., 2018), our study showed no association between smoking and mothers' behaviour towards their children. In Japan, however, children exposed to smoking had insufficient tooth brushing and SSP consumption (Tanaka et al., 2015). Nevertheless, parents' nonregular tooth brushing of their children's teeth and household smoking are risk factors for ECC development (Boustedt, Roswall, Twetman, & Dahlgren, 2018; Tanaka et al., 2015; Watanabe et al., 2014). Smokers tended to have poor oral habits, an unhealthy lifestyle, and low oral health awareness, which may also affect their children's oral health status (Tanaka et al., 2015). Smoking parents tended to brush their child's teeth less frequently, to clean their child's pacifier in their own mouth before giving it to their child, and to give their children SSB more frequently than nonsmokers (Tanaka et al., 2015).

### 4.2 Sugar consumption and oral health/general health

High sugar consumption may impair not only children's oral health but also their general health. For instance, the risk for obesity and certain systemic diseases, such as diabetes and cardiovascular diseases, increases (Matthews, Wien, & Sabate, 2011; Nadeau, Maahs, Daniels, & Eckel 2011). In recent decades, childhood overweight and obesity have become a global public health concern (Ng et al., 2014). Excessive sugar consumption has increased and starts early (Laitala et al., 2018). The relationship between high sugar intake and dental caries is well known (Moynihan & Kelly, 2014; Sheiham & James, 2014a), and studies have reported an association between childhood obesity and dental caries (Hayden et al., 2013). Caries often occur when sugar constitutes 2–3% of one's energy intake (Sheiham & James, 2014b). The World Health Organization (WHO) has been concerned with high sugar consumption and the development of dental caries and has issued the WHO guidelines for free sugars consumption: to limit free sugars intake to less than 10% of total energy intake and ideally to less than 5% (WHO Guideline, 2015). Our study showed that a high number (>70%) of children received SSP, and about 10% consumed SSB daily/frequently. This result is in line with those of earlier studies (Fan, Wang, Xu, & Zheng, 2019; Watanabe et al., 2014). Considering the intensity of SSP consumption, the findings of this study revealed that about two thirds of the children consumed up to 2–3 SSPs daily. The same pattern of SSP consumption among young children is evident in Finland, China, and Japan (Fan et al., 2019; Laitala et al., 2018; Watanabe et al., 2014). In Sweden, about one third of mothers gave beverages to their one‐year‐olds, a habit that associated with the presence of mutans streptococci (Ingemansson Hultquist, Lingström, & Bågesund, 2014).

Parents should be encouraged to control their children's cariogenic food consumption to minimise their children's risk not only of dental caries but also of obesity (Tinanoff, 2017). Poor oral health and obesity share a common background (Tinanoff, 2017). Beverages other than water between meals greatly increase the risk for ECC development and weight gain among young children (Watanabe et al., 2014). Although parents believe juice is a healthy dietary choice (Van Lippevelde et al., 2013) and a relatively high proportion of parents include it with meals (Boustedt et al., 2018), its potential to promote overweight is similar to that of SSB consumption (Van Lippevelde et al., 2013). These findings raise concerns that children are at risk for not only early childhood caries but also general health problems.

### 4.3 Children in Lithuania

Specialists recommend that from the earliest days of birth, nutrition should be healthy and meet the needs of a growing and developing baby (World Health Organization, 2015). In Lithuania, most preschool children eat according to dietary recommendations: they eat breakfast and four to five meals per day, but a minority of preschool children consume SSPs and drink SSB daily (Žalnieraitienė et al., 2018). Moreover, almost 40% of parents reported that their children's SSP consumption was too high, whereas a fifth of them believed it was insufficient (Žalnieraitienė et al., 2018). Another study revealed that the frequency of SSB intake tends to rise with age, and 16.3% of 7‐ to 8‐year‐old Lithuanian children drink SSB daily or almost daily (Petrauskienė, Žaltauskė, & Albavičiūtė, 2015). These findings show a need to raise awareness among parents in Lithuania of the potential harm caused by excessive consumption of SSP to oral and general health. In our study, a clear majority of mothers reported giving SSB less than daily or never. Healthy eating habits, such as drinking water, developed in early childhood will presumably continue throughout one's lifetime. In general, data regarding the mode of SSP consumption among young children in Lithuania is scarce, and new research is needed in this field.

The directive of the Minister of Health in Lithuania (The Order of the Minister of Health of the Republic of Lithuania, 2019a) states that family physicians or paediatricians chosen by the parents should examine children's health and psychomotor development at healthcare services. The same regulation states that a dentist or dental hygienist should perform annual dental check‐ups and provide recommendations for proper oral hygiene (The Order of the Minister of Health of the Republic of Lithuania, 2019a). Nevertheless, about one third of mothers with a young child reported not following the recommendation to brush one's teeth twice daily. This points to a need for more efforts to improve the situation in the country. Proper children's oral healthcare should be introduced already during the prenatal period. Currently, information regarding children's oral healthcare is not included in the courses given to expecting mothers at primary healthcare centres (The Order of the Minister of Health of the Republic of Lithuania, 2019b). Moreover, not only dentists but also nurses should emphasise during the children's regular general health check‐ups the importance of the parents' role in their children's oral hygiene.

Our study showed that a considerable proportion of mothers with a young child had inferior oral hygiene. Because of the social desirability effect, the true number is likely to be even higher. However, the anonymity of the participants diminished the social desirability effect. In addition, not all mothers visiting the centre completed the questionnaire, but the overall 70% response rate is acceptable. Moreover, recommended annual dental check‐ups are often postponed until the children attend day care centres with mandatory dental check‐ups (The Order of the Minister of Health of the Republic of Lithuania, 2019a). These reasons may contribute to the poor oral health of children in Lithuania. Lithuanian children with high ECC experience have severe dental problems, poor quality of life, and undergo multiple caries treatments and extractions under general anaesthesia (Jankauskiene et al., 2014). Moreover, new caries lesions often develop with insufficient oral hygiene following dental treatment under general anaesthesia (Jankauskiené, Virtanen, & Narbutaité, 2017).

## 5 CONCLUSIONS

This study revealed that about one third of mothers failed to brush their teeth twice daily, and a majority of mothers who took part in this survey did not brush their children's teeth as recommended. Mothers with a lower education and who brushed their teeth less than twice daily offered their children SSB more frequently. More emphasis on children's oral health promotion and education about the importance of proper oral hygiene habits is needed.

## 6 CLINICAL RELEVANCE

### 6.1 Scientific rationale for study

Mothers greatly influence their children's oral health. Yet, research on maternal attitudes and behaviour towards oral health is scarce in Lithuania.

### 6.2 Principal findings

A considerable number of the Lithuanian mothers did not follow the universal recommendations for tooth brushing. Mothers with a lower education and who brushed their teeth less than twice daily offered their children SSB frequently.

### 6.3 Practical implications

More emphasis needs to focus on children's oral health promotion and education.

## CONFLICT OF INTERESTS

The authors declare that they have no competing interests.

## AUTHOR CONTRIBUTIONS

S. P., J. N., and J. V. were involved in conception and design of the study. S. P. carried out data collection. S. P., J. N., and J. V. performed the analyses and interpretation of the data. All authors (S. P., J. N., A. P., and J. V.) participated in drafting and writing the manuscript. All authors read and approved the final manuscript.

## ETHICS APPROVAL

The study was approved by the Bioethics Center of the Lithuanian University of Health Sciences (No. BEC‐OF‐14). An anonymous patient characteristic form was used for data collection. Participation was voluntary and all participants provided their written consent.

## Supporting information

Data S1. The questionnaire.

## Data Availability

The datasets used and/or analysed during the current study are available from the corresponding author on reasonable request.
