# Supplementary material for: Oral health behaviour, attitude towards, and knowledge of dental caries among mothers of 0‐ to 3‐year‐old children living in Kaunas, Lithuania
Source: Clin Exp Dent Res. 2019 Dec 13;6(2):215–24. doi: 10.1002/cre2.272 (PMC7133724; doi:10.1002/cre2.272)
Supplement: Supplementary file 1 — Data S1. The questionnaire. [file CRE2-6-215-s001.pdf]

BACKGROUND:

Age of child: ____ yrs ____ months   No of children in the family: ____

Child care: ☐ home ☐ day care center ☐ family day care ☐ other

Age of mother:	☐	☐	☐	☐	☐
	≤ 24	25-29	30-34	35-39	≥ 40

Mother's education: ☐ basic ☐ secondary (professional) ☐ college ☐ university

MOTHER

Tick the correct answer to the following questions

How often do you brush your teeth?
- ☐ almost every day
- ☐ once-a-day
- ☐ more than once-a-day
- ☐ don't brush my teeth

Do you smoke?
- ☐ daily
- ☐ occasionally
- ☐ I don't smoke
- ☐ I have quitted

Tick the most suitable answer

How often do you usually eat the following products?

	≥ 3 times per day	1-2 times per day	2-5 times a week	more seldom	never
coffee or tea (with sugar)	☐	☐	☐	☐	☐
other sweet drinks	☐	☐	☐	☐	☐
buisquit, raisins or chips	☐	☐	☐	☐	☐
candy	☐	☐	☐	☐	☐

MOTHER AND CHILD

Tick the alternative best corresponding your own behavior to the following questions

	yes	no
Do you feed your child with spoon, which you have used yourself for tasting?	☐	☐
Do you have a habit to clean pacifier in your own mouth before giving it to the child?	☐	☐
Do you feed your child from plate/mug, which you have eaten/drunken from?	☐	☐
Do you kiss your child on the lips?	☐	☐

	not at all	seldom	ca. once a week	ca. every other day	every day	more than once-a-day
How often do you brush your child's teeth?	☐	☐	☐	☐	☐	☐
How often does your child get sugary drinks/juices?	☐	☐	☐	☐	☐	☐
How often does your child get sweets/candies?	☐	☐	☐	☐	☐	☐
